# Preparation of Photo-Bioelectrochemical Cells With the RC-LH Complex From *Roseiflexus castenholzii*

**DOI:** 10.3389/fmicb.2022.928046

**Published:** 2022-06-16

**Authors:** Jinsong Du, Jiyu Xin, Menghua Liu, Xin Zhang, Huimin He, Jingyi Wu, Xiaoling Xu

**Affiliations:** ^1^Photosynthesis Research Center, Hangzhou Normal University, Hangzhou, China; ^2^Department of Biochemistry and Molecular Biology, School of Basic Medical Sciences, The Affiliated Hospital of Hangzhou Normal University, Hangzhou, China

**Keywords:** photo-bioelectrochemical cell, reaction center, light harvesting complex, photosynthetic bacteria, electron transfer

## Abstract

*Roseiflexus castenholzii* is an ancient green non-sulfur bacteria that absorbs the solar energy through bacteriochlorophylls (BChls) bound in the only light harvesting (LH) complex, and transfers to the reaction center (RC), wherein primary charge separation occurs and transforms the energy into electrochemical potentials. In contrast to purple bacteria, *R. castenholzii* RC-LH (*rc*RC-LH) does not contain an H subunit. Instead, a tightly bound tetraheme cytochrome *c* subunit is exposed on the P-side of the RC, which contains three BChls, three bacteriopheophytins (BPheos), two menaquinones, and one iron for electron transfer. These novel structural features of the *rc*RC-LH are advantageous for enhancing the electron transfer efficiency and subsequent photo-oxidation of the *c*-type hemes. However, the photochemical properties of *rc*RC-LH and its applications in developing the photo-bioelectrochemical cells (PBECs) have not been characterized. Here, we prepared a PBEC using overlapped fluorine-doped tin oxide (FTO) glass and Pt-coated glass as electrodes, and *rc*RC-LH mixed with varying mediators as the electrolyte. Absence of the H subunit allows *rc*RC-LH to be selectively adhered onto the hydrophilic surface of the front electrode with its Q-side. Upon illumination, the photogenerated electrons directly enter the front electrode and transfer to the counter electrode, wherein the accepted electrons pass through the exposed *c*-type hemes to reduce the excited P^+^, generating a steady-state current of up to 320 nA/cm^2^ when using 1-Methoxy-5-methylphenazinium methyl sulfate (PMS) as mediator. This study demonstrated the novel photoelectric properties of *rc*RC-LH and its advantages in preparing effective PBECs, showcasing a potential of this complex in developing new type PBECs.

## Introduction

Photosynthesis is the process that converts solar energy into electrochemical energy and supports almost all life on Earth. It originated from bacteria, and gradually appeared in algae and higher plants through genetic transfer. Diverse photosynthetic systems have evolved to achieve higher energy transformation efficiency. The membrane-bound protein-pigment complexes in these systems are capable of catalyzing the photochemical charge separation with a quantum efficiency of close to 100% (Blankenship et al., 2011; Singh et al., 2018), which has inspired the exploration and development of the third-generation solar cells (O’Regan and Grätzel, 1991; Wang et al., 2020, 2021). The third-generation solar cells prepared by plant and bacterial photosynthetic complexes are collectively known as photo-bioelectrochemical cells (PBECs) (Feng et al., 2016; Sekar et al., 2016; Musazade et al., 2018; Zhou et al., 2019; Lee et al., 2020).

In contrast to algae, cyanobacteria and higher plants that have evolved oxygenic photosynthesis, anoxygenic phototrophs are prokaryotes with primitive photosynthetic systems. In the model organism purple bacteria, light energy is absorbed by bacteriochlorophyll (BChl) B880 bound in the light harvesting complex 1 (LH1) either directly or from the peripheral antenna LH2 and transferred to the reaction center (RC), where charge separation occurs and transforms the energy into electrochemical potentials. The RC is composed of L, M subunits that each contains five transmembrane helices, and the H subunit comprising a cytoplasmic globular domain anchored to the membrane by an N-terminal transmembrane helix (Supplementary Figures 1A,B). The RC accommodates a special pair (P) of BChls, two accessary BChls and two bacteriopheophytins (BPheos), as well as an iron and two ubiquinone (UQ) molecules for electron transfer (Supplementary Figures 1C,D). Excitation of the special pair (a dimer of coupled BChls that are arranged in a slipped face-to-face π-π-stacking, which acts as primary donor in the initial steps of the charge separation) on the P-side of the RC initiates transfer of an electron along the A-branch BChl (B_A_), BPheo (H_A_), Q_A_ quinone, and iron to Q_B_ quinone. Two electrons transferred from the charge separation fully reduces the quinone to hydroquinone, which diffuses into the membrane pool and initiates cyclic electron transfer between the RC and cytochrome *bc*_1_ to build up a proton motive force for ATP synthesis. This highly stable and relatively simple RC-LH1 complex of purple bacteria has been extensively studied to develop new type of PBECs (Ravi and Tan, 2015).

To prepare an efficient PBEC, the photosynthetic complexes are required to constantly generate electrons and continuously inject these photogenerated electrons into electrodes (Paul et al., 2020; Buscemi et al., 2021). The efficiency of the photoelectric conversion is correlated with the transfer path of electrons inside the PBEC. In most PBECs prepared from purple bacteria RC-LH1, the RC is attached to the front electrode with its P-side, which enables the Q-side and cytoplasmic domain of the H subunit exposed in the mediator. The photogenerated electrons are transferred through the mediator to the counter electrode, wherein the accepted electrons pass through an external circuit to the front electrode to reduce the excited P^+^ and form a steady-state current (Supplementary Figure 1E). However, transfer of the photogenerated electrons with a mediator often decreases the electron transfer efficiency and results in loss of the photocurrent (Takshi et al., 2010). To address this problem, Masaharu et al. connected the H subunit of RC to the electrode using a connector N-(1-Pyrenyl) Iodoacetamide, which allowed the photogenerated electrons flow out of Q_B_ and be directly transferred into the electrode (Trammell et al., 2006). Unfortunately, presence of the relatively thick H subunit (24Å) and the connector (4Å) increased the actual distance between Q_B_ and the electrode to 28Å, which resulted in a relatively low electron transfer efficiency of this system.

Beatty et al. then shortened the distance between Q_B_ and the electrode by genetically truncating the H subunit of *Rhodobacter sphaeroides*. The shortest mutant (45M RC) contains 45 amino acids left at the N-terminal of H subunit, it retained the main function of charge separation (formation of P^+^ and Q_A_^–^) but lacked a bound quinone at Q_B_ site. When the H-truncated RC (45M-M229Q_B_^–^) was attached to a gold electrode *via* a Cys engineered near the Q_A_ site, the steady-state currents increased to 200 nA/cm^2^ when using 20 mM hydroquinone as the sole mediator (Jun et al., 2018). The photocurrents were increased and decreased in about 0.2 s, a rate that is one or two orders of magnitude faster than previously reported values obtained using soluble mediators (Trammell et al., 2006; Terasaki et al., 2009). However, truncation of the H subunit increased the conformational or electrostatic changes of the quinone binding pockets and decreased the binding affinity of Q_B_ (Sun et al., 2015), causing the photogenerated electrons transferred only from Q_A_ to the electrode (Supplementary Figure 1F; Jun et al., 2018). Notably, complete removal of the H subunit from an intact RC severely reduced the electron transfer from Q_A_ to Q_B_ by approximately 10^2^ to 10^3^-fold, the resultant LM RC retained 95 ± 3% electron transfer to the primary Q_A_ (Debus et al., 1985). Consistently, the electron transfer rate from H_A_^–^ to Q_A_ was decreased by approximately 4-fold in the LM dimer (Sun et al., 2016). Therefore, a RC-LH that lacks the H subunit but maintains intact electron transfer capacity is required to enhance the electron transfer efficiency of the PBECs.

**FIGURE 1:**
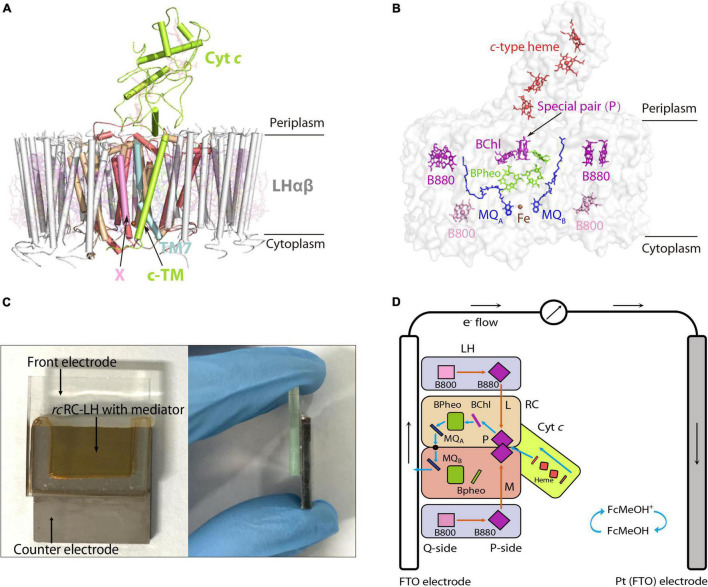
Preparation of an FTO-Pt PBEC using the RC-LH from *R. castenholzii (rcRC-LH) rc*. **(A)** Overall structure of the *rc*RC-LH (PDB ID: 5YQ7) is shown at the side view. The light harvesting (LH) complex (white) is composed of 15 LHαβ heterodimers in the form of an opened elliptical ring, which encircles the L (wheat), M (salmon), and cyt *c* subunit (limon) composed RC. The tetraheme (red sticks) binding domain of the cyt *c* subunit is exposed at the periplasmic side. Especially, a novel transmembrane helix of the cyt *c* (c-TM) inserts into the gap of the LH ring, and form a quinol shuttling channel in together with a newly identified X subunit (pink). An unassigned TM7 transmembrane helix (light cyan) is identified near the transmembrane helices of the L and M subunits in the RC. All the cofactors are shown as stick models with a transparency at 80%. **(B)** Spatial organization of the pigments and electron carriers in the *rc*RC-LH. Each LHαβ non-covalently binds two B880s (purple) at the periplasmic side, one B800 (pink) at the cytoplasmic side. The RC accommodates a special pair of BChls (P, purple), an accessary BChl (purple), and three BPheos (chartreuse), as well as an iron (brown sphere) and two menaquinone-11 (MQ, blue) molecules. The four *c*-type hemes bound in the cyt *c* subunit are exposed in the periplasmic side. All the cofactors except the iron are shown as stick models. **(C)** Construction of the *rc*RC-LH based FTO-Pt PBEC. The PBEC is composed of a FTO glass as the front electrode and a Pt-coated FTO glass as the counter electrode, a mixed solution of *rc*RC-LH with the mediator is injected into the cavity between the two electrodes as the electrolyte. A front (left) and side (right) view of the apparatus is shown. **(D)** Diagram of the operating mechanism of the *rc*RC-LH prepared FTO-Pt PBEC. The electron donor FcMeOH (hydroxymethylferrocene) was used as the mediator. Orange arrows indicate the route of light energy transfer, and blue arrows indicate the route of electron transfer. The P-side represents the direction of the *rc*RC-LH wherein the special pair (P) is located, and the direction where the MQ_A_ and MQ_B_ located is named the Q-side. The photogenerated electrons released from the excited P^+^ are transferred along the accessary BChl, BPheo to Q_A_, an iron, then to Q_B_. The electrons at the Q_B_ directly enter the front electrode (FTO) and are transferred to the counter electrode (Pt), wherein the accepted electrons pass through the *c*-type hemes *via* the mediator to reduce the excited P^+^ and form a steady-state current.

*Roseiflexus castenholzii* (*R. castenholzii*) is an ancient green non-sulfur bacteria that contains a unique photosynthetic system. In contrast to purple bacteria, *R. castenholzii* does not contain a peripheral LH2. It contains only one LH that encircles the L, M, and cyt *c* subunits composed RC to form an opened elliptical ring (Yamada et al., 2005; Majumder et al., 2016; Xin et al., 2018). Especially, *R. castenholzii* RC-LH (*rc*RC-LH) lacks the H subunit, it tightly associates a cytochrome (cyt) *c* subunit through a novel transmembrane helix (c-TM), with the tetraheme binding domain protruding into the periplasmic space, which can accept electrons transferred from the periplasmic electron donors. The c-TM further inserts into the gap of the LH ring, in together with a newly identified transmembrane helix X to form a quinol shuttling channel (Figure 1A and Supplementary Figure 2A). The RC contains three BChls and three BPheos (Collins et al., 2010), instead of four BChls and two BPheos in purple bacteria. Additionally, two menaquinone-11 (MQ) molecules have been identified within the RC, instead of the UQs bound in purple bacteria RC (Figures 1A,B and Supplementary Figures 1A–D). These novel structural features of *rc*RC-LH are beneficial for enhancing the electron transfer efficiency and subsequent photo-oxidation of the *c*-type hemes (Deisenhofer and Michel, 1989). Importantly, absence of the H subunit in *rc*RC-LH provides a prerequisite for direct transfer of the photogenerated electrons from the Q-side to the electrodes. However, the photochemical properties of *rc*RC-LH and its applications in developing PBECs have not been characterized.

In this study, we made the first attempt to prepare a PBEC using the *rc*RC-LH complex (Figures 1C,D). The extracted *rc*RC-LH showed excellent photoreduction activity in catalyzing the reduction of the electron acceptor methylviologen (MV^2+^). The PBEC was prepared using an overlapped fluorine-doped tin oxide (FTO) glass as the front electrode, a Pt-coated glass as the counter electrode, and the purified *rc*RC-LH mixed with a mediator as the electrolyte. Photoelectric measurements characterized the immobilization of *rc*RC-LH on the electrode, the electron transfer path, and photocurrent intensities of the PBECs using different mediators. The results of this study revealed the photochemical properties and potentials of *rc*RC-LH in preparing an effective PBEC, and will contribute to the development of new type PBECs in the future.

## Materials and Methods

### Extraction and Purification of the *rc*RC-LH Complex

The *rc*RC-LH complex was extracted and purified from photoheterotrophically grown *R. castenholzii* as previous reported (Xin et al., 2018). The homogeneity and purity of the complex was monitored at each stage of the preparation by recording the absorption spectrum from 250–900 nm. The final 880 nm to 280 nm absorption ratio for the purified *rc*RC-LH complex was above 1.25.

### Measurement of the Stability and Photoreduction Activity of the *rc*RC-LH Complex

To test the stability of the purified *rc*RC-LH complex, light energy was simulated with a 200 W incandescent lamp. The freshly prepared *rc*RC-LH complex was placed 40 cm away from the light source and exposed under light for 12 h at 25 °C. Then, the absorption spectrum over the range 700–1000 nm before and after the illumination was measured *via* UV-Vis spectrometry (Mapada P6, China). The photoreduction activity of the *rc*RC-LH was measured by mixing the *rc*RC-LH complex (20 μM) with 20 mM methylviologen (Aladdin Chemical Reagent Co., Ltd., United States), 0.2 mM polyvinyl pyrrolidone (Macklin Biochemical Co., Ltd., United States), 35 mM L-Lysine (Biosharp Life Sciences Co., Ltd., China), and 500 mM 2-Hydroxy-1-ethanethiol (Aladdin Chemical Reagent Co., Ltd., United States) to a final volume of 1 mL. Using deionized water as a negative control group, the absorbance of the mixed solution at 605 nm was recorded with and without light exposure. All the measurements were performed independently three times. The average and standard deviations of the values were calculated.

### Preparation of an FTO-Pt PBEC Using the *rc*RC-LH Complex

We prepared the FTO-Pt PBECs according to previous literatures (Tan et al., 2012a,b). The FTO conducting glass (TEC 7 ohm/sq, 20 mm square × 2.2 mm thick; Shangzhuo Technology Co., Ltd., China) was dipped into acetone and isopropanol successively for 30 min and dried to serve as the front electrode. The counter electrode was comprised of a second piece of FTO conducting glass covered with a layer of 25 nm-thick Pt that was deposited by magnetron sputtering technology (MSP-300C, China). The conductive film of the two electrodes was checked with scanning electron microscope (Hitachi S-4800, Japan), and the water contact angles of the surfaces were measured using Drop Shape Analyser (Kruss DSA100, Germany). The two electrodes were then connected by a hot melt adhesive film (300 μm-thick; Xingxia Polymer Products Co., Ltd., China) to form a cavity of about 1 cm^2^ between the boundaries of the two electrodes. A 30 μL electrolyte solution containing 40 μM of the *rc*RC-LH complex and varying mediators (each at 250 μM concentration) such as hydroxymethylferrocene (FcMeOH), methylviologen, N, N, N′, N′-Tetramethyl-p-phenylene-diamine dihydrochloride (TMPD), and 1-Methoxy-5-methylphenazinium methyl sulfate (PMS) (Aladdin Chemical Reagent Co., Ltd., United States) was injected into the cavity. Finally, the opening was sealed by hot glue gun.

### Characterizations of the Photoelectric Properties of the FTO-Pt PBEC

To measure the photostability of the prepared FTO-Pt PBEC, light energy was simulated with a 200 W incandescent lamp. The FTO-Pt PBEC was placed 40 cm away from the light source and exposed under light for 12 h at 25 °C. Then, the absorption spectrum of *rc*RC-LH in FTO-Pt PBEC over the range 700–1000 nm before and after the illumination was measured *via* UV-Vis spectrometry. The prepared FTO-Pt PBEC was exposed to a light source that was simulated using a 200 W incandescent lamp at a distance of 40 cm. The photocurrent intensities in the cell were measured by an electrochemical workstation (Lanlink LK2005A, China), with an applied voltage at 0 mV and the current intensity in the dark environment as the baseline. Since the active areas of the cells were slightly different, an opaque mask with a 0.1 cm^2^ hole was placed on the outer surface of front electrode before measurement, which ensured that the intensity of the incident light in each cell reached approximately 1.5 mW/cm^2^.

## Results

### The Cofactors Arrangement and Electron Transfer Routes in the *rc*RC-LH Complex

In our previously reported cryo-EM structure of *rc*RC-LH (Xin et al., 2018), the only LH is composed of 15 LHαβ heterodimers in the form of an opened elliptical ring. Each LHαβ non-covalently binds two B880s at the periplasmic side, one B800 at the cytoplasmic side, and one keto-γ-carotene spanning the interface between LHαβ for light harvesting and transfer (Figure 1A and Supplementary Figures 2A,B). The L and M subunits of RC accommodate a special BChl dimer P (B865), a BChl (B818), and three BPheos, as well as an iron and two MQ-11 molecules that are positioned within 14Å of each other (Figure 1B and Supplementary Figure 3A), a distance necessary for efficient electron orbital coupling and energy resonance (Osyczka et al., 2004). The distance between the special pair (P) and the nearest *c*-type heme is approximately 10.4Å (Supplementary Figure 3A), which is adequate for the subsequent reduction of the excited P^+^, through accepting the electrons transferred back from the four *c*-type hemes.

The light energy absorbed by the coupled pigments (B800, B880, and keto-γ-carotene) in LH are transferred to the special pair (P) of the RC. Once excited, the state P^+^ is stabilized by transferring an electron to the primary electron acceptor, BChl, within several picoseconds, and then passed through BPheo, MQ_A_, and iron to MQ_B_ (P^+^Q_B_^–^) (Supplementary Figure 3B). P^+^ can be reduced by electrons transferred back from the *c*-type hemes. Transfer of two electrons from the photochemical charge separation forms P^+^Q_A_^–^Q_B_^–^, which is fully reduced to hydroquinone (QH_2_) after accepting the second photogenerated electron and incorporating two protons from the cytoplasm (Supplementary Figure 3B). The tightly bound tetraheme cyt *c* subunit endows the *R. castenholzii* RC a much higher efficiency for the energy transfer and photo-oxidation of the *c*-type hemes, which is significantly different from the soluble and monoheme cyt *c* in purple bacteria.

### The Stability and Photoreduction Activity of the *rc*RC-LH Complex

The *rc*RC-LH complex was extracted and purified from the heterotrophically grown *R. castenholzii.* Spectroscopic analyses revealed typical Qy bands of the LH-bound BChls at 800 nm (B800) and 880 nm (B880), and a Qx band of BChls at 594 nm. The RC-bound BPheos were determined at 760 nm, and the carotenoids showed absorption at 457, 482, and 519 nm. Near this region, an absorption peak corresponding to the oxidized cyt *c* was observed at 410 nm, as well as the split peaks of BChls at 374 nm (Supplementary Figure 4A). The absorption spectrum of *rc*RC-LH barely changed after exposure to simulated light energy for 12 h, indicating the excellent photo-thermal stability of the complex (Supplementary Figure 4B).

**FIGURE 2:**
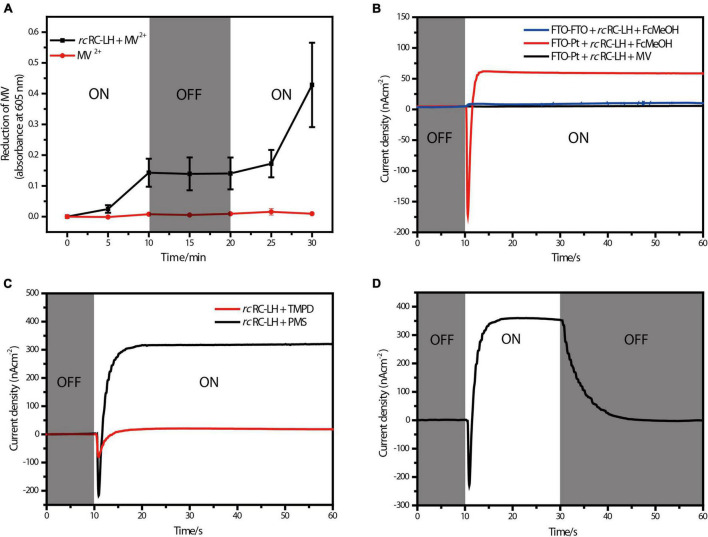
Photoelectric characterizations of the FTO-Pt PBEC. **(A)** Photoreduction activity of the *rc*RC-LH complex measured by using MV^2+^ as the electron acceptor, the absorbance of MV^+^ at 605 nm was recorded in the presence and absence of the *rc*RC-LH. The illumination period is indicated in the white sections of graph, and the dark period is indicated in gray. **(B)** Measurements of the photocurrent intensities of alternative PBECs. The photocurrent intensities were plotted over time with (white background) or without (gray background) illumination. One PBEC was prepared using FTO-glass as both electrodes (FTO-FTO), the other PBEC was prepared with only FTO-glass as the front electrode (FTO-Pt). FcMeOH (hydroxymethylferrocene) and MV (methylviologen) were used as the mediators in these PBECs. **(C)** Comparison of the photocurrent intensities of the FTO-Pt PBECs prepared using TMPD and PMS as the mediators. White graph background indicates a period of illumination, gray represents the period of darkness. **(D)** Changes of the photocurrent intensities of the PMS FTO-Pt PBEC upon alternating illumination conditions. White graph background indicates a period of illumination, gray represents the period of darkness.

Methylviologen (MV^2+^) is an electron acceptor that can be easily reduced to MV^+^ upon accepting a single electron (Wang et al., 2021). Using 2-Hydroxy-1-ethanethiol as the electron donor and MV^2 +^ as the electron acceptor, we measured the photochemical activity of *rc*RC-LH through recording the absorbance of MV^+^ at 605 nm (Figure 2A). In presence of the *rc*RC-LH, the absorbance of MV^+^ at 605 nm continually increased under illumination and kept constant when the illumination was turned off. Once re-exposed to the simulated light, the absorbance quickly increased, indicating that the *rc*RC-LH was excited by the light energy to initiate charge separation and subsequent electron transfer, releasing electrons to reduce MV^2+^ to MV^+^. Therefore, the extracted *rc*RC-LH possessed considerable photoreduction activity *in vitro*.

### Preparation of an FTO-Pt PBEC Using the *rc*RC-LH Complex

To prepare PBECs, the photosynthetic complex is often fixed to the working electrode by physical adsorption or chemical coupling after a long period of incubation with the electrode (Tan et al., 2012a). Before preparation, the working electrode usually needs to be pre-processed by high-temperature sensitization, chemical coupling, or modification of the surfaces (Lebedev et al., 2008; Terasaki et al., 2009). Here, a piece of FTO conducting glass deposited with a 350 nm-thick conductive film of fluorine-doped tin oxide was dipped into acetone and isopropanol successively for 30 min, then dried to serve as the front electrode. The surface of the FTO-glass is roughly textured (Supplementary Figure 5A), as a result of the low symmetric tetragonal crystal structures of fluorine-doped tin oxide (Zhang et al., 2018). This structure is similar to the wrinkles on the surface of the bacterial plasma membrane, which facilitates attachment of the photosynthetic complexes (Csiki et al., 2018).

The counter electrode is comprised of a second piece of FTO conducting glass covered with a layer of 25 nm-thick Pt, which can evenly cover the surface of the FTO-glass without affecting its electrical conductivity (Supplementary Figure 5B). Moreover, coating of the Pt layer also enabled the two electrodes to show distinct wettability (Supplementary Figures 5C,D). Notably, the surface of the front electrode is hydrophilic with a water contact angle of 45.1°, whereas the Pt surface of the counter electrode is more hydrophobic (with a contact angle of 91.6°), which would affect the attachment of the *rc*RC-LH onto the electrodes. An electrolyte containing 40 μM *rc*RC-LH and alternative mediator was injected into the cavity formed between the two electrodes to prepare the FTO-Pt PBEC (Figures 1C,D). The absorption spectrum of the *rc*RC-LH stored in the FTO-Pt PBEC were barely changed after exposure to the simulated light energy for 12 h (Supplementary Figure 4C), indicating a moderate photostability of the FTO-Pt PBEC.

By constructing a PBEC containing two electrodes with distinct wettability, the *rc*RC-LH was expected to selectively adhere to the hydrophilic surface of the front electrode (Tan et al., 2013). To test which electrode the *rc*RC-LH was attached to, we constructed two PBECs using the electron donor hydroxymethylferrocene (FcMeOH) as the mediator. One PBEC was constructed using FTO-glass for both electrodes (FTO-FTO), and the other was constructed with FTO-glass as the front electrode only (FTO-Pt). The current intensities of the two PBECs were measured *via* an electrochemical workstation under illumination. Without illumination, the current intensities of both FTO-FTO and FTO-Pt were zero. However, the photogenerated current of the FTO-Pt was much higher than that of FTO-FTO under illumination (Figure 2B), indicating the *rc*RC-LH was selectively adhered to the hydrophilic surface of the FTO glass, and that it was active in catalyzing the photochemical conversion reactions to generate electrons at the front electrode under illumination. In the FTO-FTO PBEC, the photogenerated electrons were probably being neutralized, due to the comparable binding capacity of the *rc*RC-LH complex onto the two electrodes with the same wettability. Therefore, we successfully immobilized the *rc*RC-LH complex onto the hydrophilic front electrode of the FTO-Pt PBEC through controlling the wettability of the surfaces instead of utilizing a complicated pre-processing procedure, thus eliminating a time- and resource-consuming step.

### Photoelectric Characterizations of the FTO-Pt PBEC

When using electron donor FcMeOH as the mediator, a reverse current spike was generated once the PBEC was exposed to illumination (Figure 2B), indicating the photogenerated electrons were flowing out of the front electrode. The current spike was then deflected to form a steady-state current after about a 5 s illumination (Figure 2B), indicating the electrons were flowing into the front electrode from the *rc*RC-LH. The appearance of the reverse current spike was intriguing, as in the PBECs prepared from purple bacteria RC-LH1, the reduction rate of P^+^ is usually thousands of times higher than the oxidation rate of Q_B_^–^ (Agalidis and Velthuys, 1986; Tan et al., 2012a). During photochemical electron transfer of the *rc*RC-LH prepared FTO-Pt PBEC, the excited P^+^ is reduced by the electrons transferred from the four *c*-type hemes *via* the mediator FcMeOH (Figure 1D). As the rate of electrons flow more quickly into the P-side than out of the Q-side, the electron carriers in the RC serve as an electron sink. The over-oxidized FcMeOH extracts electrons from the front electrode, generating a reverse current spike. When the electron carriers in the RC are fully occupied and FcMeOH^+^ is spreading inside the PBEC to reach an equilibration, the photogenerated electrons continuously flow into the front electrode to form a steady-state current (Figure 1D).

To allow the photogenerated electrons directly flow into the electrode, the Q_B_ of the RC are required to face the electrode (Page et al., 1999; Winkler and Gray, 2014; Jun et al., 2018). Therefore, attachment orientation of the RC-LH on the electrode is essential for direct electron transfer into the front electrode. In the PBECs prepared by connecting the P-side of RC-LH1 to the front electrode (Supplementary Figure 1E), the mediator MV^2+^ accepted the photogenerated electrons from the Q-side, and transferred the electrons to the counter electrode to form a steady-state current (Kondo et al., 2007). To check the attachment direction of *rc*RC-LH on the electrodes, we replaced the electron donor FcMeOH with electron acceptor MV^2+^. As shown in Figure 2B, when using MV^2+^ as a mediator, the FTO-Pt PBEC did not generate a current under illumination, indicating that no photogenerated electrons were transferred to the counter electrode *via* MV^2+^. Since the tetraheme binding domain of the cyt *c* subunit is exposed on the P-side of the *rc*RC-LH, if it is attached to the front electrode with the P-side, Q_B_^–^ would reduce the electron acceptor MV^2+^ to ensure continuous photocurrent generation. Therefore, lost of the photocurrent indicated that the *rc*RC-LH complex was selectively attached to the front FTO electrode with its Q-side. In contrast to the previously reported PBECs, the photogenerated electrons in our FTO-Pt PBEC can directly enter the front electrode without assistance of a mediator, avoiding the current losses that result from mediated electron transport.

The mediator also plays a significant role in the electron transfer of the PBECs, as it continuously supplies electrons to the excited P^+^ through the *c*-type hemes, in order to ensure continuous photocurrent generations (Figure 1D). Both N, N, N′, N′-Tetramethyl-p-phenylene-diamine dihydrochloride (TMPD) and 1-Methoxy-5-methylphenazinium methyl sulfate (PMS) can serve as electron donors for the *rc*RC-LH. The vacuum potentials of TMPD and cyt *c* are −4.7 eV, whereas PMS, with a vacuum potential of −4.5 eV, is more favorable to provide electrons for the cyt *c* (Yaghoubi et al., 2014). As shown in Figure 2C, when TMPD and PMS were used as mediators, a steady-state current was generated in 10 s after illumination. The efficiency of the solar cell is strongly dependent on the short-circuit photocurrent density (*J*_SC_) and the associated open-circuit voltage (*V*_OC_). Specifically, the PMS PBEC could reach a steady-state *J*_SC_ of 320 nA/cm^2^ and *V*_OC_ of 50 mV, which is much higher than that generated by the TMPD PBEC. These results indicated that the photocurrent intensities of the PBEC can be increased by changing the vacuum potentials of the mediator. In addition, the photocurrent of PMS PBEC started to decrease and gradually reached 0 nA/cm^2^ when the light was turned off (Figure 2D). The gradual decrease of the photocurrent was a result of the flowing of the stored photogenerated electrons into the electron carriers of the RC, when the reduction rate of P^+^ is much higher than the oxidation rate of Q_B_^–^.

## Discussion and Future Perspective

In this study, we prepared a PBEC using the RC-LH complex from an ancient green non-sulfur bacteria *R. castenholzii*, which are phylogenetically distant from other anoxygenic photosynthetic bacteria. Different from the well-studied purple bacteria, *R. castenholzii* does not have a peripheral antenna LH2, instead it contains only one LH, which is composed of 15 αβ-polypeptides to form an opened elliptical ring. The open conformation of the LH ring structurally resembles that of the purple bacteria LH1, which also contain an LH1 ring with gap produced by PufX, protein-W, or special transmembrane helices (Qian et al., 2021a,b; Swainsbury et al., 2021). However, the c-TM and the newly identified X in *rc*RC-LH share no sequence identity and spatial organizations with any of these proteins. Compared to the monomeric RC-LH1 of *Rba. Sphaeroides*, both c-TM and X are located at different positions with distinct conformations (Figure 1A and Supplementary Figures 1A,B). Specifically, the c-TM and X are almost parallel with the LHαβ heterodimers, whereas the PufX is tilted with the LHαβs at approximately 60°, and the protein-U is formed by two transmembrane helices in a U-shape (Figure 1A and Supplementary Figures 1A,B; Qian et al., 2021b). Besides, incorporation of the B800s at the cytoplasmic side of *rc*LH combines the spectroscopic properties of purple bacteria LH1 and LH2, which are beneficial for enhancing the light harvesting efficiency of the *rc*RC-LH.

Compared to the LH that contains different compositional and structural features, the core components of the RC, including the L, M-subunits and cofactors are structurally conserved with that of purple bacteria (Supplementary Figures 1A–D, 2A,B). Notably, purple bacteria usually contain an H subunit that is important for regulating the assembly and electron transfer of the RC (Debus et al., 1985; Sun et al., 2015; Sun, 2017; Cao et al., 2022). However, presence of the H subunit often increased the distance between Q-side of the RC and the electrode, and decreased the electron transfer efficiency in PBECs. In contrast, absence of the H subunit allows the *rc*RC-LH to be selectively adhered to the hydrophilic surface of the front electrode with its Q-side, then the photogenerated electrons directly enter the electrode without assistance from any mediators (Figure 1D). Moreover, *R. castenholzii* RC contains a membrane-bound cyt *c* subunit that is tightly associated with the L and M subunits, with its tetraheme binding domain exposed at the P-side (Figure 1A and Supplementary Figure 2A). Exposure of the tetraheme binding domain endows *rc*RC-LH a higher photo-oxidation rate of the *c*-type hemes, which accepted the electrons transferred from the counter electrode and reduced the excited P^+^ to form a continuous photocurrent (Figure 1D). Therefore, the novel structural features of *rc*RC-LH provide a prerequisite for constructing effective PBECs.

The PBEC in this study was prepared using overlapped FTO glass and Pt-coated glass as electrodes, and the *rc*RC-LH mixed with a mediator as the electrolyte. As shown in Figure 2C, when using PMS as the mediator, the PBEC produced continuous photocurrent in 10 s after illumination (Figure 2C). A steady-state *J*_SC_ of up to 320 nA/cm^2^ was generated with *V*_OC_ at 50 mV and the incident light intensities at 1.5 mW/cm^2^. In the FTO-Pt PBEC prepared using *Rba. sphaeroides* RC-LH1 (Supplementary Figure 1E), generation of the steady-state current took about 20 s after illumination. However, the steady-state *J*_SC_ of this PBEC could reach 900 nA/cm^2^ for the fresh PMS, with a *V*_OC_ of 80 mV and the incident light intensities at 10 mW/cm^2^ (Tan et al., 2012b). In respect to the power conversion efficiency that is calculated from the product of the *V*_OC_ and *J*_SC_ divided by the incident light intensities, a higher power conversion efficiency of 1.07 × 10^–3^% was obtained for the *rc*RC-LH based PBEC than that prepared by *Rba. sphaeroides* RC-LH1 (7.2 × 10^–4^%). Therefore, although the *rc*RC-LH prepared FTO-Pt PBEC generated lower *V*_OC_ and *J*_SC_, but it achieved a relatively faster and higher power conversion efficiency than the RC-LH1 based FTO-Pt PBEC.

Difference of the power conversion efficiency are mainly resulted from the distinct structural features and attachment orientations of the *rc*RC-LH and RC-LH1. It has been shown that removal of the H subunit from purple bacteria RC results in enhanced exposure of the semiquinone sites in the LM dimer (Sun, 2017), indicating the cytoplasmic domain of the H subunit shields the internal quinones of the RC-LH1. Therefore, presence of the H subunit hindered electron transfer from the Q-side of the RC to the electrode and decreased the electron transfer efficiency in the RC-LH1 prepared PBECs, no matter the RC-LH1 was attached to the electrode with its P-side or Q-side (Supplementary Figures 1E,F). Genetic truncation of the cytoplasmic domain of the H subunit indeed shortened the distance between Q_B_ and electrode to about 12Å, a steady-state *J*_SC_ of up to 200 nA/cm^2^ was generated when using hydroquinone as the mediator (Jun et al., 2018). However, the photogenerated electrons were transferred only from Q_A_ to the electrode (Supplementary Figure 1F), since the Q_B_ binding affinity was dramatically decreased due to truncation of the H subunit (Sun et al., 2015). In contrast, the *rc*RC-LH is a natural protein complex bound with endogenous menaquinones, the conformations of the quinone binding pockets were not affected during preparation of the PBECs. As a result, the photogenerated electrons could transfer from the excited P^+^ to both the Q_A_ and Q_B_ in the PBECs. Therefore, absence of the H subunit in *rc*RC-LH is an advantageous structural feature that not only effectively exposes the Q-side of the RC for direct electron transfer to the electrode, but also enhances the power conversion efficiency of the PBECs.

However, the steady-state *V*_OC_ and *J*_SC_ of the *rc*RC-LH prepared FTO-Pt PBEC are relatively low, as compared with the dye-sensitized solar cells that commonly generate *V*_OC_ at several hundreds of mV and *J*_SC_ in mA/cm^2^ range. The redox potential difference between the electrolyte and the photo-oxidized BChls in the RC (P/P^+^) is a major determinant of the *V*_OC_. In *Rba. Sphaeroides* RC-LH1 prepared PBEC, the *V*_OC_ scaled approximately linear with the measured potential of the electrolyte. On the other side, the *J*_SC_ was also increased by the vacuum potentials of the mediator. The FTO-Pt PBECs containing fresh TMPD (−4.73 eV) typically produced steady *V*_*OC*_ of approximately 7 mV and *J*_SC_ of 150 nA/cm^2^, which are much lower than that generated by the fresh PMS (−4.51 eV) (Tan et al., 2012b). Consistently, both the *V*_OC_ and *J*_SC_ of the *rc*RC-LH prepared PBEC were remarkably increased when PMS was used as the mediator (Figure 2C). Therefore, it is possible to increase the *V*_OC_ and *J*_SC_ of the *rc*RC-LH based PBECs by simple manipulation of the electrolyte or alternating the potential of the coupled P/P^+^ through engineering of the RC. In addition, the photocurrent output can also be improved by optimizing the coverage and assemble form of the photoactive proteins on the electrode, exploiting the use of different electrode materials, fabrication procedures, and alternative incident light intensities of these PBECs. Besides, the electrolyte in our FTO-Pt PBEC could still have the loss of volatilization during long-time exposure to the light. Therefore, a more stable electrolyte with minimal volatilization rate also needs to be investigated in the future.

## Conclusion

We prepared a novel FTO-Pt PBEC that converts the simulated light energy into steady-state current, using the natural *rc*RC-LH complex as the photovoltaic module. In contrast to the purple bacteria RC-LH1, the *rc*RC-LH does not contain the H subunit. Instead, a tightly bound tetraheme cyt *c* subunit is exposed on the P-side of the RC, which allows the *rc*RC-LH to selectively adhere onto the hydrophilic surface of the front electrode with its Q-side. As a result, the photogenerated electrons can directly enter the front electrode without assistance of a mediator, avoiding decrease of the electron transfer efficiency and loss of the photocurrent. Additionally, the photocurrent intensities of this FTO-Pt PBEC can be enhanced by changing vacuum potentials of the mediators. A steady-state current of up to 320 nA/cm^2^ was generated in the presence of PMS as the mediator. The results of this study provide a new perspective for preparing new type PBECs using the unique structural features of the *rc*RC-LH complex.

## Data Availability Statement

The original contributions presented in this study are included in the article/Supplementary Material, further inquiries can be directed to the corresponding author.

## Author Contributions

XX initiated the project and supervised all experiments. JD prepared the PBECs and characterized its photoelectrical properties. JX and ML purified the RC-LH complex from *R. castenholzii*. XZ, HH, and JW assisted in measurement of the photoelectric properties of the *rc*RC-LH based PBECs. XX and JD analyzed the data and wrote the manuscript. All authors contributed to the article and approved the submitted version.

## Conflict of Interest

The authors declare that the research was conducted in the absence of any commercial or financial relationships that could be construed as a potential conflict of interest.

## Publisher’s Note

All claims expressed in this article are solely those of the authors and do not necessarily represent those of their affiliated organizations, or those of the publisher, the editors and the reviewers. Any product that may be evaluated in this article, or claim that may be made by its manufacturer, is not guaranteed or endorsed by the publisher.
