# Supplementary material for: Preparation of Photo-Bioelectrochemical Cells With the RC-LH Complex From Roseiflexus castenholzii
Source: Front Microbiol. 2022 Jun 16;13:928046. doi: 10.3389/fmicb.2022.928046 (PMC9243436; doi:10.3389/fmicb.2022.928046)
Supplement: Supplementary file 1 [file Data_Sheet_1.pdf]

Supplementary Material for

Preparation of Photo-Bioelectrochemical Cells With the RC-LH Complex From *Roseiflexus castenholzii*

Jinsong Du[1], Jiyu Xin[1,2], Menghua Liu[1], Xin Zhang[1], Huimin He[1], Jingyi Wu[1], Xiaoling Xu[1,2*]

[1] Photosynthesis Research Center, Hangzhou Normal University, 311121, Hangzhou, China

[2] Department of Biochemistry and Molecular Biology, School of Basic Medical Sciences, and The Affiliated Hospital of Hangzhou Normal University, 311121, Hangzhou, China

*** Correspondence:** Xiaoling Xu (xuxl@hznu.edu.cn)

Supplementary Figures

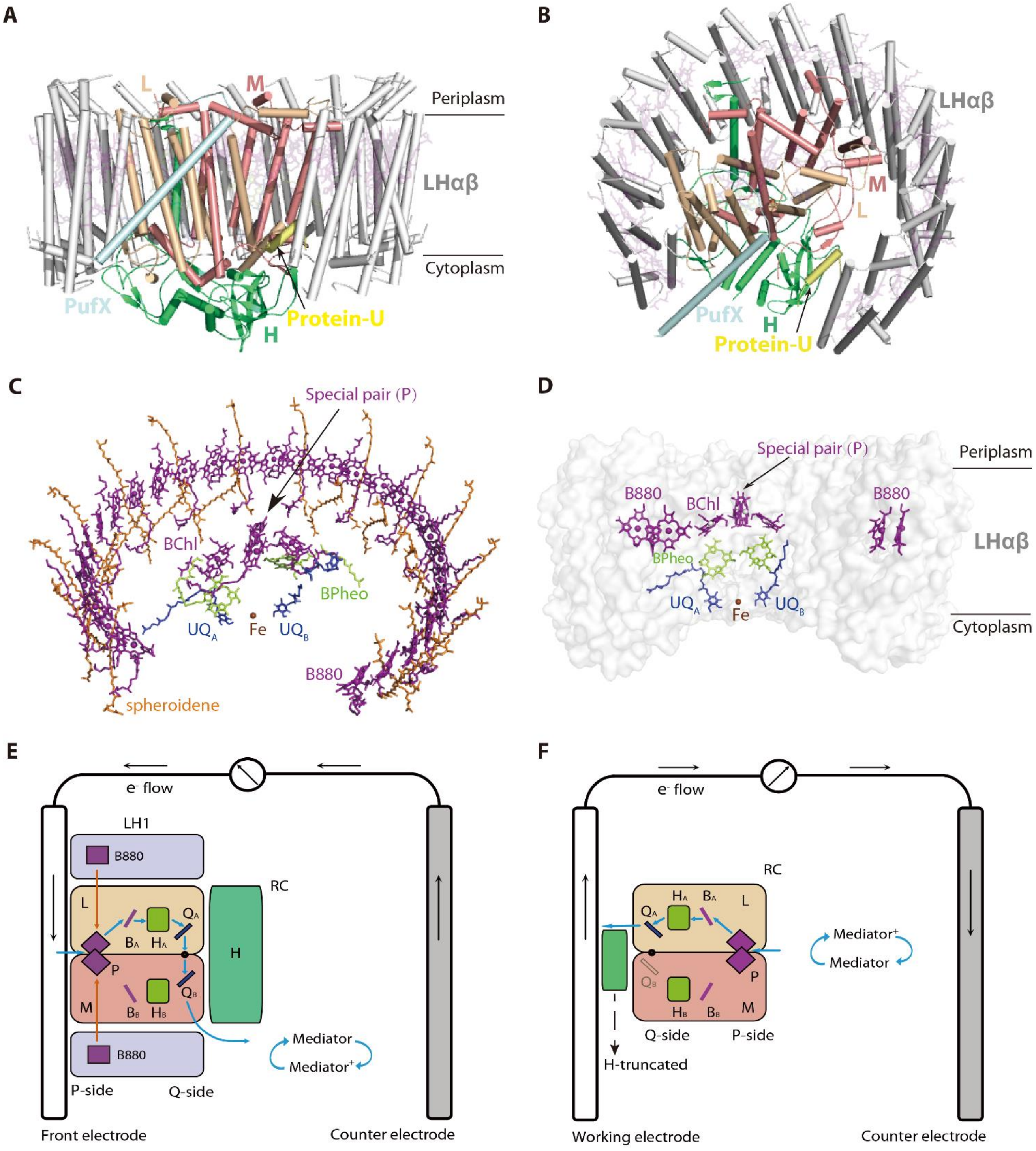

Supplementary Figure 1. Overall structure of the RC-LH1 from purple bacteria *Rhodobacter sphaeroides*, and the operating mechanisms of the PBECs prepared by the intact RC-LH1 and H-truncated RC. **(A, B)** Overall structure of the monomeric RC-LH1 from *Rba. sphaeroides* (PDB ID: 7F0L) is shown at the side (A) and bottom-up (B) view. The LH1 is composed of 14 αβ-polypeptides (white) to form a C-shaped structure with a large ring opening. The RC is composed of the L (wheat)

and M (salmon) subunits that each contains five transmembrane helices, and the H subunit (lime green) that contains a single transmembrane helix and a cytoplasmic globular domain. A previously unrecognized protein-U (yellow) is located opposite the PufX (light cyan) near the LH1-ring opening. **(C)** Arrangement of the cofactors in the RC-LH1 complex. The LH1 binds 28 BChls *a* (purple) and 26 carotenoid spheroidene (orange) to form an opened pigments ring, whereas the RC accommodates a special pair (P) of BChls (purple), two accessary BChls (purple) and two BPheos (chartreuse), an iron (brown sphere) and two ubiquinone (UQ, blue) molecules. All the pigments are shown as stick models. **(D)** Spatial organization of the electron carriers in *Rba. sphaeroides* RC. The RC-LH1 is shown as surface in white, the electron carriers in the RC and two representative B880 pairs in the LH1 are shown in stick models. **(E)** Diagram of the operating mechanism of the PBECs prepared by the intact RC-LH1. Orange arrows indicate the route of light energy transfer, and blue arrows indicate the route of electron transfer. The RC-LH1 is attached on the front electrode with its P-side, with the cytoplasmic domain of H subunit exposed in the electrolyte. The photogenerated electrons are transferred along the accessary BChl (B_A), BPheo to Q_A, an iron, then to Q_B, the electrons are transferred through a mediator to the counter electrode, wherein the electrons pass through an external circuit to the front electrode to reduce the excited P^+ and form steady-state current. **(F)** Diagram of the operating mechanism of the PBECs prepared by the H-truncated RC from *Rba. sphaeroides* (*Photosynth. Res*., 2018, 137(2): 227-239). The H-truncated RC is adhered to the working electrode (gold) with its Q-side, which allows the photogenerated electrons entering the electrode without a mediator. However, truncation of the H subunit decreased the binding affinity of Q_B quinone, causing the electrons transferred only from Q_A to the working electrode.

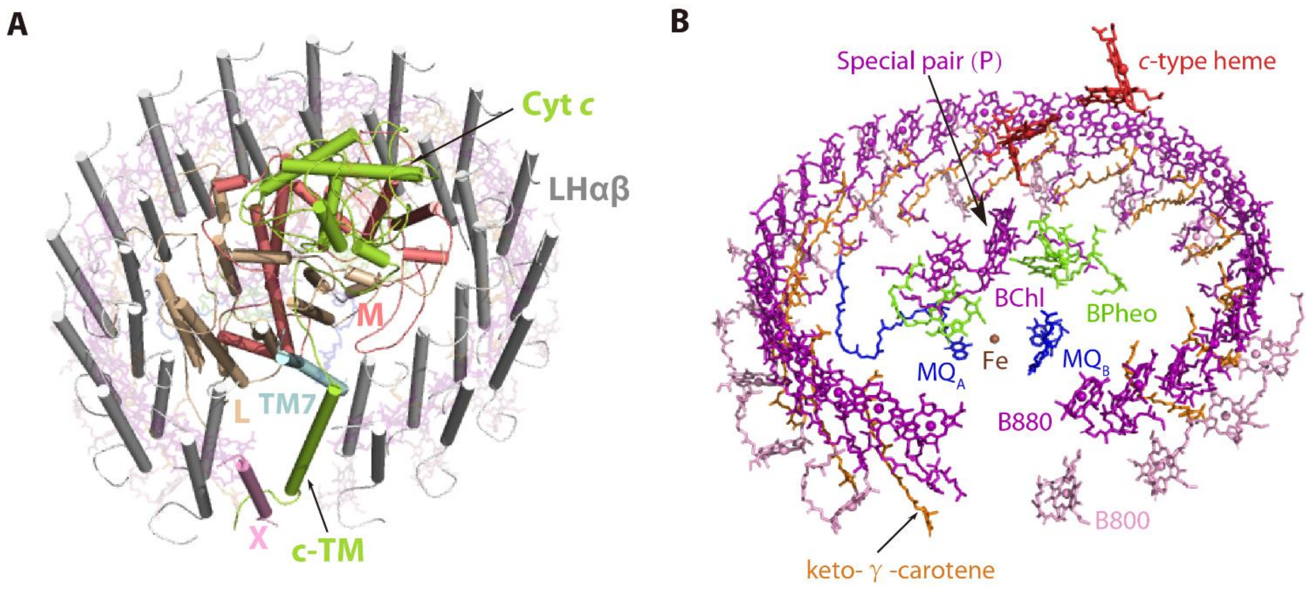

Supplementary Figure 2. Overall structure of *R. castenholzii* RC-LH (*rc*RC-LH). **(A)** Overall structure of the *rc*RC-LH (PDB ID: 5YQ7) is shown at the bottom-up view. The only LH is composed of 15 LHαβ heterodimers in the form of an opened elliptical ring, which encircles the L (wheat), M (salmon), and cyt *c* subunit (limon) composed RC. The tetraheme (red sticks) binding domain of the cyt *c* subunit is exposed at the periplasmic side. Especially, a novel transmembrane helix of the cyt *c* (c-TM) inserts into the gap of the LH ring, and form a quinol shuttling channel in together with a newly identified subunit X (pink). An unassigned TM7 transmembrane helix (light cyan) is identified near the transmembrane helices of the L and M subunits in the RC. All the cofactors are shown as stick

models with a transparency at 80%. **(B)** Arrangement of the cofactors in the *rc*RC-LH. Each LHαβ noncovalently binds two B880s (purple) at the periplasmic side, one B800 (pink) at the cytoplasmic side, and one keto-γ-carotene (orange) spanning the interface between LHαβ. The RC accommodates a special BChl dimer P (purple), an accessary BChl (purple), and three BPheos (chartreuse), as well as an iron (brown sphere) and two menaquinone-11 (MQ, blue) molecules. The four *c*-type hemes (red sticks) of the cyt *c* subunit are exposed in the periplasmic side. All the pigments are shown as stick models.

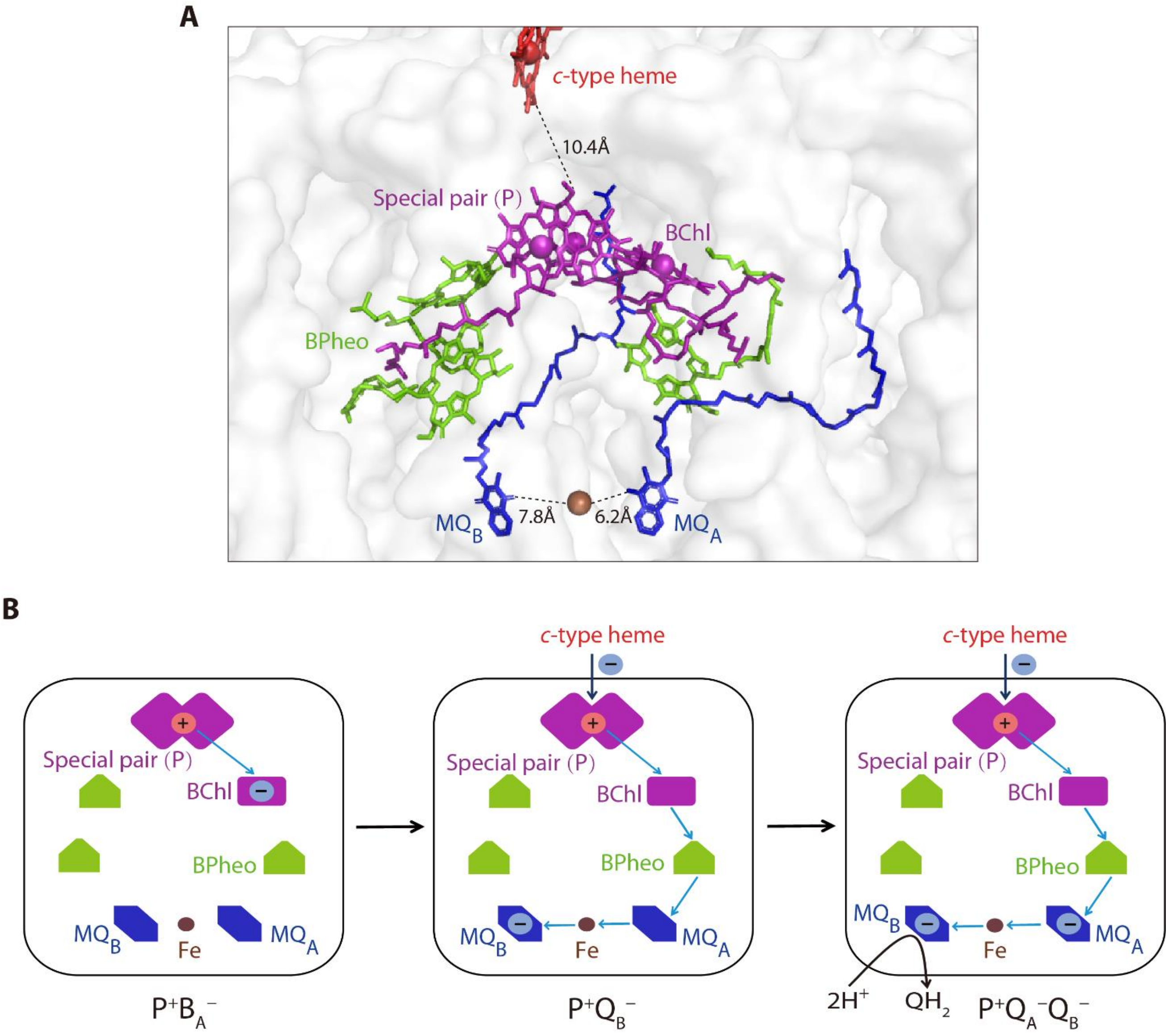

Supplementary Figure 3. Overview of the bound electron carriers **(A)** and proposed charge separation mechanism **(B)** of *R. castenholzii* RC. The electron carriers in the RC are shown in stick models with color codes for all panels: purple, BChls; tv-red, *c*-type heme; chartreuse, BPheos; blue, menaquinones (MQ); brown, iron. The distances between some electron carriers are indicated as dashed lines and labeled. P represents the special pair, it is a dimer of coupled BChls that are arranged in a slipped face-to-face π-π-stacking, acting as primary donor in the initial steps of the charge separation. $P^+B_A^-$ indicates the electrons are extracted from the special pair (P) and transferred to B_A, in which state the

P^+ carries a positive charge after releasing an electron and B_A^- is negatively charged upon accepting an electron. $P^+Q_A^-Q_B^-$ represents the state that the electrons are extracted from the special pair (P) and transferred to MQ_A and MQ_B, in which MQ_A^- and MQ_B^- carry negative charges upon accepting the electrons.

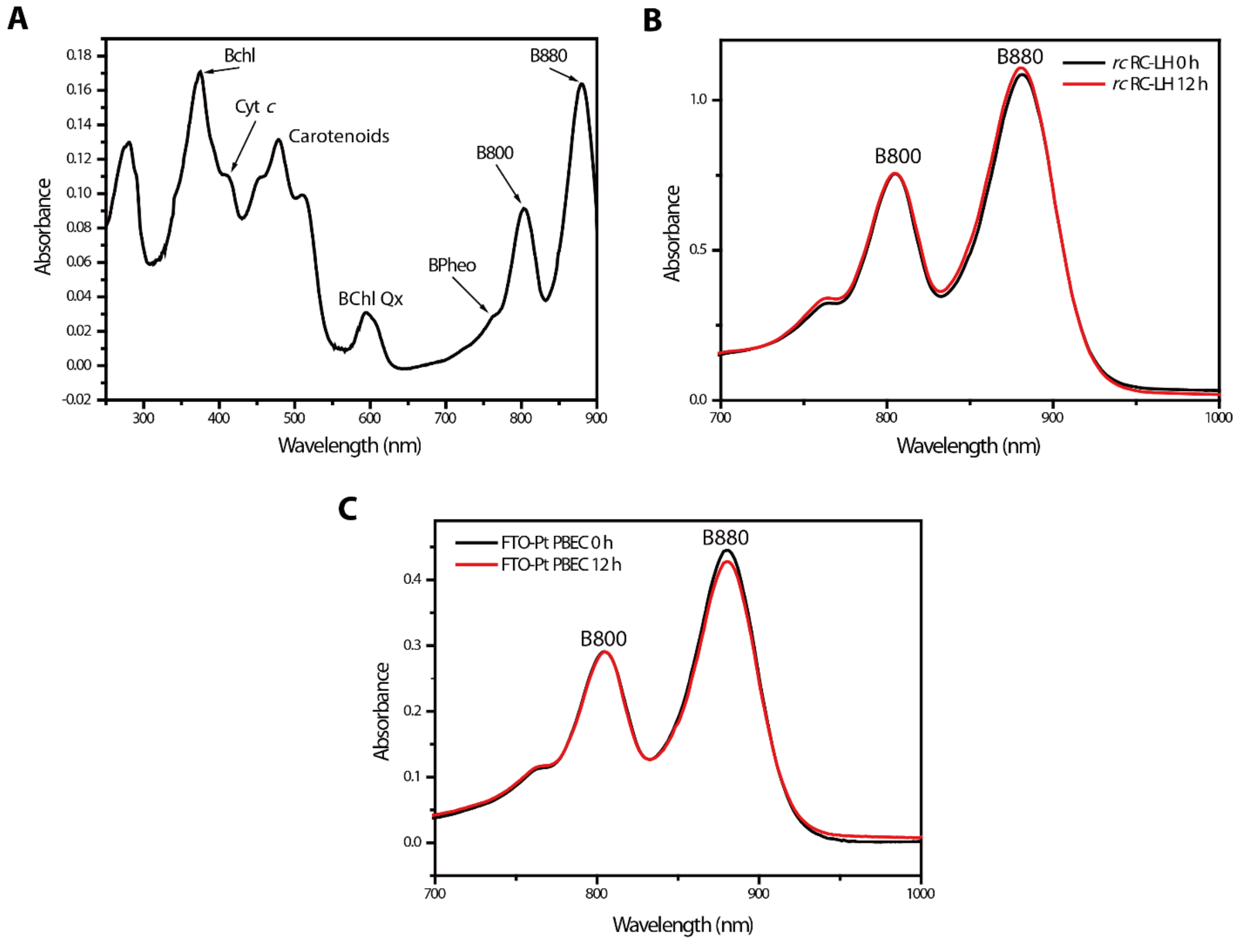

Supplementary Figure 4. Absorption spectrum and photo-stability of the purified *rc*RC-LH complex. **(A)** Absorption of the purified *rc*RC-LH complex was recorded over a range of 250 - 900 nm. The characteristic Qy bands for the B800 and B880, Qx band for BChls, and the absorption peaks for the carotenoids and cyt *c* are labeled. **(B)** The UV-Vis spectrum of *rc*RC-LH complex before and after a 12 h exposure under the simulated light energy at 25 °C. **(C)** The UV-Vis spectrum of *rc*RC-LH stored in the FTO-Pt PBEC before and after a 12 h exposure under the light environment at 25 °C.

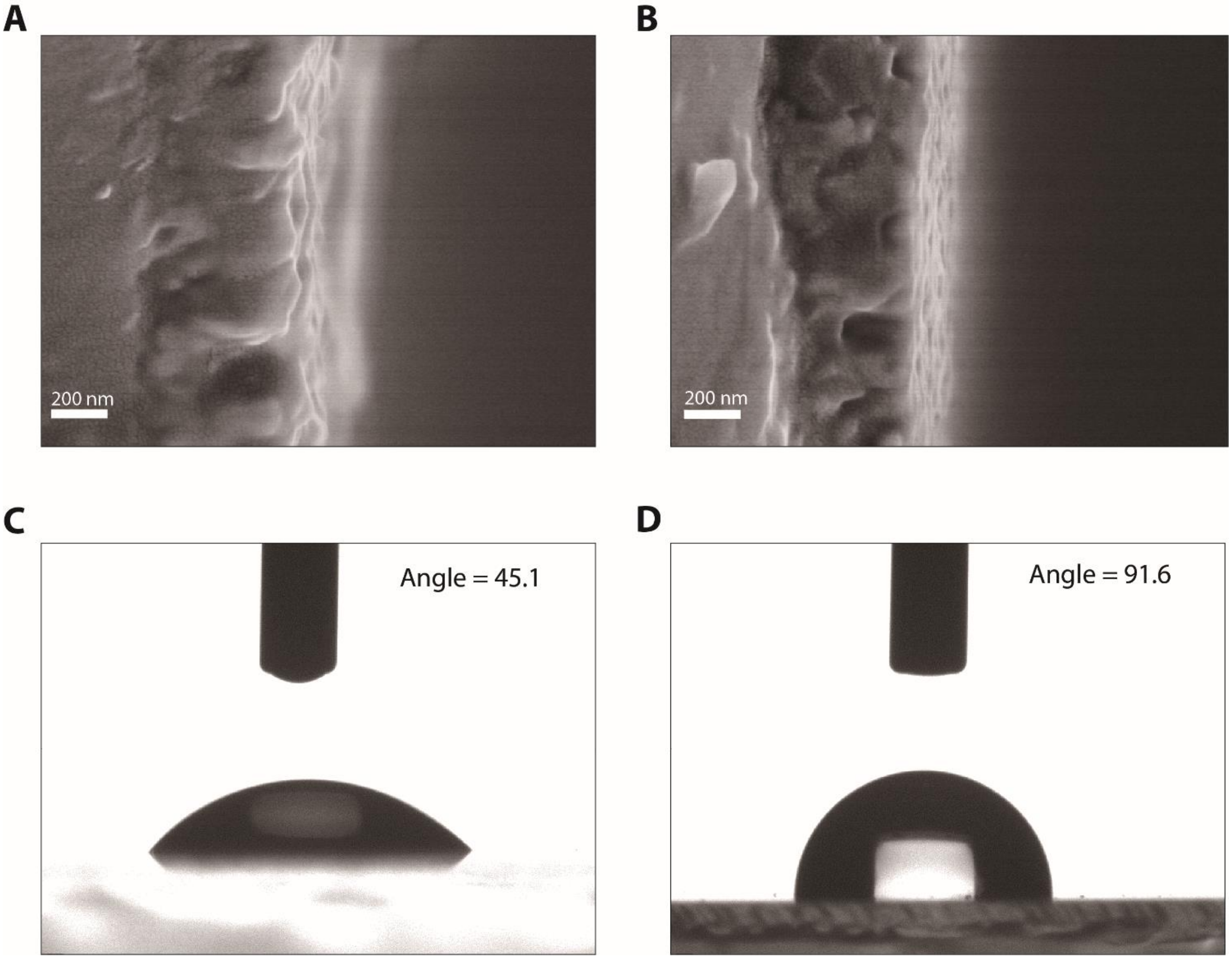

Supplementary Figure 5. (A) Scanning electron microscope (SEM) observations of the side of the FTO conducting glass. The fluorine-doped tin oxide film deposited on the glass is about 350 nm-thick, and showed relatively rough textures due to the low symmetric tetragonal crystal structure of tin oxide. **(B)** SEM observations of the side of the Pt-coated glass. After coating with a layer of 25 nm-thick Pt, the FTO conductive film became more planar with no change in thickness. **(C)** The water contact angle of the FTO conducting glass is 45.1° on the surface. **(D)** The water contact angle of the Pt-coated glass on the surface. The water contact angle of the FTO increased to 91.6° after being coated with Pt, resulting in a more hydrophobic surface.
